# Conscious intention: a challenge for AIR theory

**DOI:** 10.3389/fpsyg.2015.00675

**Published:** 2015-05-20

**Authors:** Myrto Mylopoulos

**Affiliations:** ^1^Department of Cognitive Sciences, Institute Jean NicodParis, France; ^2^Philosophy and Institute of Cognitive Science, Carleton UniversityOttawa, ON, Canada

**Keywords:** attention, intention, air theory, consciousness, working memory

A primary goal of any theory of consciousness is to provide an informative account of what makes the difference between conscious and nonconscious mental states. Typically, whether or not a given theory is successful in this regard is measured with respect to its ability to explain what it is for paradigmatically sensory states, for example, visual, auditory, or somatosensory states, to be conscious. And it is often assumed, either implicitly or explicitly, that whatever account is supplied for such states can be safely generalized to accommodate other types of mental state as well.

Here I challenge this assumption as it relates to Jesse Prinz's (2000, 2012) Attended Intermediate-level Representation (AIR) theory of consciousness. In particular, I raise doubts about whether the theory in its present form can account for conscious intentions, largely stemming from a pair of its core commitments. First, the AIR theory has it that “consciousness arises when and only when intermediate-level representations are modulated by attention” (Prinz, 2012, p. 89). Second, Prinz defends the view that “all consciousness is perceptual” (Prinz, 2012, p. 150). In other words, all conscious states are sensory states. So, the AIR theorist is faced with the following dilemma: either conscious intentions are intermediate-level sensory representations to which we can attend or, despite appearances to the contrary, our intentions are never conscious. I'll present some reasons to be skeptical of the viability of both of these options, which, taken together, suggest that the AIR theory does not, as it stands, have the resources to explain conscious intentions.

Before getting to these concerns, though, it will be useful to clarify what Prinz means by “intermediate-level,” “sensory,” and “attended” representations. An intermediate-level representation is defined relative to high-level and low-level representations in terms of the degree of specificity of its content. So, for example, in vision, high-level states represent categorical features of objects in a viewpoint-invariant way, whereas low-level states represent detailed local features of objects like edges and orientation, and intermediate-level states represent object features such as boundaries and contours from a specific viewpoint. When such states are modulated by attention, they are made available to working memory for further processing, which allows these representations to be used in further capacities like verbal report and reasoning. Finally, a sensory state for Prinz refers to a state with a representational format that is specific to a sensory modality, which is itself construed as a dedicated input system (see also Prinz, 2007).

Given these parameters, why not simply view conscious intentions as attended intermediate-level sensory states? Indeed, Prinz (2012) pursues a parallel strategy in order to explain how it is that we can have conscious thoughts, even though thoughts are themselves high-level states. He maintains that thoughts are conscious as long as they are “encoded in sensory vehicles and have no qualities above and beyond their sensory qualities” (p. 151). In particular, Prinz holds that a thought can be “rendered conscious” by forming an intermediate-level sensory image of what it represents, and attending to *that* state. So, for example, my thought that Paris is beautiful in the spring is conscious when I form a sensory image of, say, the Eiffel Tower and the sun shining upon it. Analogously, perhaps the AIR theory might accommodate conscious intentions by viewing them as states that become conscious when one forms and attends to an appropriately related sensory image.

This proposal may seem attractive, but it faces some serious difficulties. For one, we sometimes form conscious intentions without any accompanying sensory imagery. Upon realizing that I am out of milk, I might form an intention to go to the store later without visualizing my future action. Nonetheless, I might report my intention and use it in the service of further practical reasoning to plan out the rest of my day. On just about every other theory of consciousness, the AIR theory included, only conscious states are accessed and utilized in this way. So it seems that I can have a conscious intention without its being encoded in any sensory vehicle.

But perhaps one will not be moved by this concern because, one may insist, even if some of our conscious intentions are unaccompanied by visual imagery, they *are* accompanied by “inner speech,” which is properly viewed as a form of verbal imagery, and thus sensory imagery. I find this suggestion implausible. Sometimes we consciously intend to do something without having yet put the intention into words, even in inner speech. But even if one denies this, there is the further problem, that Prinz recognizes, of capturing the *attitudinal* component of a conscious intention via such verbal imagery. Rendering conscious the content of an intention by way of verbal imagery may be possible, but we also need a way to explain consciously *intending* to do something rather than, say, consciously *predicting* that we will do it, or consciously *desiring* to do it, which content-wise may look the same. Verbal imagery is not up to this task.

Prinz's (2012) solution here is to appeal to emotions for differentiating, in a sensory way, the attitudes of our conscious mental states. Applying this to the case of desire, Prinz writes: “If I want it to be the case that my candidate wins, I will feel nervous anticipation, and the thought of victory will instill delight, while the thought of defeat will usher in waves of despair. On experiencing any of these fluctuating feelings, I may report that I desire a victory” (p. 164). But our conscious intentions do not have a signature emotional profile that we can appeal to in order to determine that we are intending to do something as opposed to desiring to do it. To be clear, I am not here denying that intentions involve or are accompanied by affective or motivational qualities—perhaps that's true. Nor am I denying that such qualities would be properly construed as sensory qualities—perhaps that's true as well. The worry, rather, is that whatever sensory affective or motivational qualities they may involve or be accompanied by will not suffice to distinguish an intention to do something from a desire to do it, since a desire will be accompanied by those very same types of qualities.

In addition, it's worth stressing that even if these worries were successfully addressed, the present proposal would still not actually explain how a nonconscious intention becomes a conscious intention. Any conscious sensory images corresponding to the content of nonconscious intentions would plainly be distinct states from those intentions, since the intentions themselves are not sensory images. (This holds true for Prinz's treatment of conscious thoughts as well.) But then the nonconscious intention itself would still fail to be conscious, and so it is difficult to see how this proposal helps with our initial challenge. Indeed, this worry equally applies to Prinz's account of conscious thought.

Another general strategy available to Prinz, as mentioned, is to deny that intentions are ever conscious, and to hold instead that we are in error whenever we take ourselves to be consciously intending. Indeed, Prinz seems sympathetic to this idea, explicitly entertaining the view that, “… we are not directly aware of action decisions, and to that extent, conscious will is an illusion” (p. 199). On this proposed view, we never form conscious intentions prior to action, but we engage in *post-hoc* reconstructive inferences that convince us that we do.

As evidence for this hypothesis, Prinz discusses some experimental work carried out by Lau et al. (2007). The authors used a Libet-style (see Libet et al., 1983) paradigm, asking participants seated in front of a clock to report the time at which they became aware of deciding to act. They then applied transcranial magnetic stimulation (TMS) over the pre-supplementary motor area either immediately after the action was performed or 200 msec after. The surprising result was that participants' judgments of when they first became aware of deciding to act were shifted backwards in time on the TMS trials. In other words, when TMS was administered, they reported being aware of deciding to act at an earlier time compared with trials where no TMS was administered. Lau et al. (2007) conclude that, “… the perceived onset of intention depends, at least in part, on neural activity that takes place after the execution of action” (p. 81). On the basis of these results, Prinz speculates that, “[f]or all we know, conscious decisions […] may arise after actions have taken place and then get erroneously backdated to earlier points in time” (p. 198).

But it's not clear how this would help with the present issue, since Prinz would still be faced with the task of explaining what it means for a conscious decision to arise after the action. I have already raised concerns for viewing such states as attended intermediate-level sensory states, and it's not clear what other options are available to the AIR theorist.

Of course, Prinz need not be wedded to this version of the reconstructive hypothesis. He has the option of saying instead that no conscious decision ever arises, even after the action, just the reconstructive inference. But this version of the reconstructive hypothesis is not supported by the Lau et al. (2007) results. It may be that our timing judgments related to intentions can be influenced by events occurring after the action, but while this may suggest that we are sometimes wrong about precisely when we actually form intentions to act—something that would hardly be surprising given how rare such judgments are—it provides no evidence whatsoever for the claim that we are sometimes wrong about whether we formed an intention *at all*, at *some point* prior to the action. To bring this point home, it is worth point out that analogous findings of subjective timing distortions pertaining to the onset of sensory experiences, among them Libet's own (see Libet et al., 1979), provide no evidence that such experiences do not take place, or that we are sometimes wrong in reporting that they occurred. Nor are they taken to provide such evidence. There is plainly a difference between a judgment that a mental event occurred at time t and a judgment that a mental event occurred at all. And we have no reason to be skeptical of our ability to make the latter type of judgment based on the Lau et al. (2007) results.

Prinz has a reply available to him here, for there is one putative source of evidence for the reconstructive hypothesis that I have yet to address, and this comes from the work of the late psychologist Daniel Wegner. One of Wegner's most widely discussed studies is his “I Spy” study (Wegner and Wheatley, 1999; Wegner, 2002), which appears to show that we can also sometimes be confused into thinking that we intend an action that we do not intend. This would help bolster the claim that, not only are we sometimes wrong about the timing of our intentions, but about whether or not we intended to do something in the first place.

Briefly, in Wegner and Wheatley's (1999) study, participants were seated across from a confederate, and asked to jointly move a computer mouse—in a “ouija board” type set-up—that controlled a cursor on a computer screen positioned nearby, on which was displayed a number of objects. The participants and the confederate would move the cursor around the screen together and, after approximately 30 s, they were instructed to stop and rate the extent to which they felt they intended the stop to on a scale ranging from 0 to 100 (“0” indicating “I allowed the stop to happen” and “100” indicating “I intended to make the stop”). What the participants didn't know is that on some trials the confederate was in full control of the object that the cursor stopped upon. Nonetheless, when participants heard the name of the target object over headphones just before these stops, they were more likely to give higher ratings on the scale. The authors conclude that they “perceived the forced stops as intended” (p. 489).

But, as many have at this point stressed (Nahmias, 2005; Shepherd, 2013; Mylopoulos and Lau, 2014), this conclusion is not supported by the results of the experiment—so much so that it is somewhat surprising how often this study is cited as evidence for the reconstructive hypothesis. Perhaps the strongest objection to this interpretation is that, on average, the participants *barely rated the forced stops as over halfway between being allowed and being intended*. This strongly suggests that they did not view themselves as having consciously intended the stops but were, if anything, uncertain about their causal contributions to the stops. And this is hardly unexpected given the highly ambiguous context within they were asked to act.

In sum, I think there is a significant challenge here for the AIR theorist. It does not seem that conscious intentions are sensory states, and it does not seem that we have any reason to deny that they exist. But if so, then the AIR theory cannot account for all conscious mental states.

## Conflict of interest statement

The author declares that the research was conducted in the absence of any commercial or financial relationships that could be construed as a potential conflict of interest.
